# Enhanced Ethanol Degradation Using *Geobacter metallireducens* *–* *Shewanella oneidensis* Coculture Bioanodes in Single-Chamber Microbial Electrolysis Cells

**DOI:** 10.1021/acsomega.6c10207

**Published:** 2026-10-28

**Authors:** Avinash Jukanti, Vamshi Krishna Bommagani, Abhishiktha Chiliveru, Alex Schechter, Rivka Cahan

**Affiliations:** † Department of Chemical Engineering, 42732 Ariel University, Ariel 40700, Israel; ‡ Department of Chemical Sciences, 42732 Ariel University, Ariel 40700, Israel; § Research and Development Centre for Renewable Energy, New Technologies, Research Centre (NTC), University of West Bohemia, Pilsen 30100, Czech Republic

## Abstract

Ethanol-rich effluents from biorefineries, distilleries, and fermentation industries are abundant but remain difficult to valorize because alcohol oxidation rarely aligns with the extracellular electron-transfer (EET) capacity of single exoelectrogenic species. A coculture bioanode combines *Geobacter metallireducens* (GM), which carries high-flux c-type cytochrome EET but modest alcohol dehydrogenase activity, with *Shewanella oneidensis* MR-1 (SO). The coculture was operated in single-chamber microbial electrolysis cells (MECs) for 82 days. The MEC was enriched first with sodium acetate (SA, 1600 mg L^–1^) and then with the transitional feed (800 mg L^–1^ SA + 5 mM ethanol, followed by 5 to 20 mM ethanol). The GM+SO coculture outperformed both monocultures in current, with the hierarchy GM+SO > GM > SO preserved across every substrate phase. LSV current density reached 1.66 ± 0.27 A m^–2^ in GM+SO under 5 mM ethanol, representing 2.7- and 8-fold enhancements over GM and SO, respectively. GM+SO achieved the fastest degradation rate when ethanol was used as the carbon source, at 4.68 ± 0.55 mM d^–1^; it retained ethanol-removal capacity at 20 mM when GM collapsed under substrate inhibition. End point 16S rRNA sequencing showed that *Geobacter* and *Shewanella* accounted for 45% and 20% of reads in the GM+SO biofilm. PICRUSt2 predicted greater potential for alcohol and aldehyde dehydrogenases and c-type cytochrome functions in the coculture, consistent with a hypothesized pathway in which both partners oxidize ethanol to acetate and *Geobacter* dominates electron delivery to the anode. Thus, the benefit of coinoculation depends on substrate and loading, and substrate removal alone is a poor guide to partner selection for ethanol-treating bioanodes.

## Graphical abstract

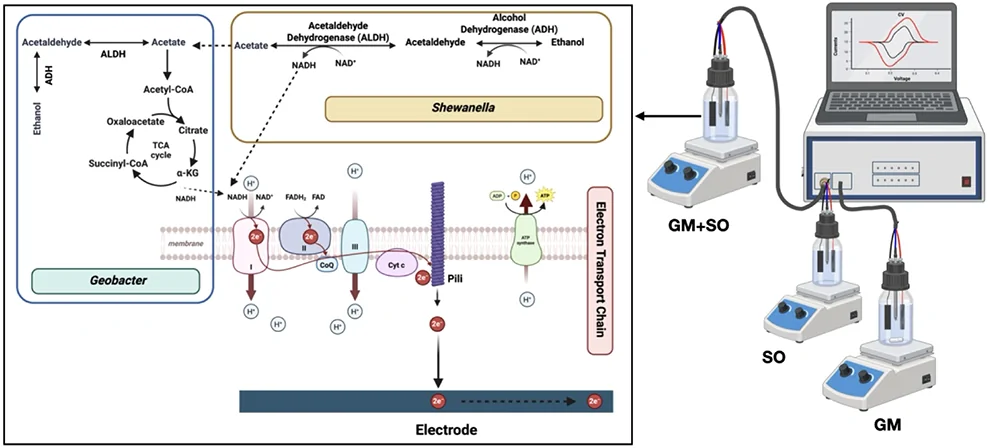

## 1 Introduction

Ethanol-bearing effluents are produced in enormous volumes by the fermentation, biorefinery, and distillery industries, where each liter of ethanol manufactured generates roughly 1–12 L of high-strength wastewater enriched in residual alcohols, organic acids, sugars, and recalcitrant melanoidins.^1,2^ Without adequate treatment, these effluents harm aquatic and terrestrial ecosystems.^3^ Conventional anaerobic digestion can recover part of the chemical energy as methane, but the process is slow, sensitive to organic shock loads, and still requires costly post-treatment of COD-rich effluent.^4^

Bioelectrochemical systems (BESs), including microbial electrolysis cells (MECs), offer an alternative route. In these systems, electroactive bacteria that oxidize organic substrates at the anode and transfer the liberated electrons through an external circuit to a cathode. With a modest applied voltage (typically 0.2–0.8 V), those electrons drive hydrogen, methane, acetate, or other cathodic products^5–7^ Acetate-fed MECs now routinely produce anodic current densities of several A m^–2^, and pilot-scale MECs have been operated on real domestic and industrial wastewaters for months.^8^ Acetate, however, is an unusually favorable substrate, and performance measured on acetate often overestimates how a bioanode will behave on other feedstocks.^9,10^ Ethanol illustrates the problem. Its conversion to a fully oxidizable intermediate is rarely matched to the extracellular electron transfer (EET) capacity of the dominant electroactive taxa, so current output often falls short of what substrate removal would predict.

We selected *Geobacter metallireducens* and *Shewanella oneidensis* MR-1 because their strengths and weaknesses are complementary with respect to this mismatch: one is an efficient electron donor to electrodes, and the other is a versatile oxidizer of alcohols. *G. metallireducens* forms dense, conductive anodic biofilms and transfers electrons directly to electrodes through outer surface multiheme c-type cytochromes and conductive filaments.^11,12^ It is also one of the very few *Geobacter* species that can oxidize ethanol. Ethanol-fed *G. metallireducens* cocultures provided the founding evidence for direct interspecies electron transfer (DIET).^13,14^ Its alcohol oxidation, however, is less efficient than its acetate oxidation.^15^ *S. oneidensis* MR-1 carries broad set of NAD^+^-dependent alcohol and aldehyde dehydrogenase enzymes and can rapidly oxidize ethanol, lactate, formate, and a range of fermentation intermediates.^16^ Its weakness lies in electron delivery. It forms only thin anodic biofilms and depends largely on secreted flavins as soluble shuttles between its outer-membrane MtrC/OmcA cytochromes and the electrode.^17^ Pure-culture *Shewanella* bioanodes, therefore, consistently underperform in side-by-side comparisons with *Geobacter* bioanodes.^18,19^ Pairing the two organisms could, in principle, couple rapid substrate activation to efficient electron delivery.

*Geobacter*-*Shewanella* cocultures have been examined in BESs before. Most of these studies used *G. sulfurreducens* and acetate or lactate as electron donors, on which neither partner is kinetically limiting.^18,20,21^ In a mixed *G. sulfurreducens*-*S. oneidensis* MFC, *Geobacter* made up about 90% of the anodic community and *Shewanella* less than 10%.^22^ Other long-term studies suggest the minor partner can still influence current generation despite its low abundance.^18,23^ Alcohol-fed mixed cultures have been tested mainly with glycerol or short transient feeds.^24,25^ To our knowledge, no study has examined a defined *G. metallireducen* *s* + *S. oneidensis* bioanode under sustained ethanol feeding at increasing concentrations. How such a pair processes ethanol therefore remains unresolved: through acetate cross-feeding, flavin-mediated transfer, direct electrical contact, or a combination of these. In an MEC, both organisms can reach the electrode directly, so they need not exchange electrons with each other. Complementary EET routes coordinated by metabolite exchange are an equally plausible arrangement.

In this study, single-chamber MECs inoculated with *G. metallireducens* (GM), *S. oneidensis* MR-1 (SO), and their coculture (GM+SO) were operated for 82 days. Operation began with an acetate acclimation phase, followed by a stepwise ethanol series (5–20 mM). Current generation, voltammetric behavior, substrate removal (GC, HPLC, and ^1^H NMR), anodic biofilm metabolic activity, community composition, and predicted functional potential were compared across configurations. We hypothesized that ethanol is oxidized to acetate by the dehydrogenase capacity of both partners. We further hypothesized that the acetate is then oxidized by *G. metallireducens*, which delivers electrons through its cytochrome network, while *Shewanella*-derived flavins provide an additional mediated route to the electrode. This study defines the ethanol loading range over which a defined coculture sustains both substrate removal and current generation, including loadings that inhibit *G. metallireducens* alone, providing a basis for designing electroactive consortia for ethanol-rich industrial effluents. This application-driven approach questions whether coinoculation helps when the bioanode must oxidize ethanol at increasing loadings over long-term operation. Specifically, we tested (i) whether the coculture outperforms the additive expectation from its members, and whether this depends on the substrate, and (ii) whether coinoculation extends the ethanol loading range over which removal and current generation are maintained.

## 2 Methods and Materials

### 2.1 Bacterial Strains, Media, and Inoculation

*Geobacter metallireducens* GS-15 (DSM 7210) was obtained from the Leibniz Institute DSMZ (Braunschweig, Germany), and *Shewanella oneidensis* MR-1 (ATCC 700550) was obtained from the ATCC (Manassas, USA). *G. metallireducens* was cultivated anaerobically in DSMZ medium 579 (pH 6.8), containing per liter: ferric citrate, 13.70 g; ammonium chloride (NH_4_Cl), 1.50 g; sodium phosphate monobasic (NaH_2_PO_4_), 0.60 g; potassium chloride (KCl), 0.10 g; sodium acetate (CH_3_COONa), 2.50 g; sodium bicarbonate (NaHCO_3_), 2.50 g; modified Wolin’s mineral solution, 10 mL; and Wolin’s vitamin solution (Table S1), 10 mL. Ferric citrate was first dissolved in distilled water by gentle heating, and the pH was adjusted to 6.0, after which all remaining components except NaHCO_3_ and vitamins were added. The medium was sparged with nitrogen/carbon dioxide (N_2_:CO_2_) (80:20, v/v) for 30–45 min, NaHCO_3_ was then added, and the pH was adjusted to 6.8 under the same gas atmosphere. The medium was dispensed into anoxic serum vials, autoclaved (121 °C, 20 min), and filter-sterilized vitamin solution was added aseptically prior to use.

*S. oneidensis* MR-1 was cultivated aerobically in Tryptic Soy Broth (TSB; HiMedia, India) at 30 g L^–1^ (pH 7.3 ± 0.2), and autoclaved at 121 °C for 20 min. Cultures were grown at 30 °C with shaking at 150 rpm until the midexponential phase (OD_600_ ≈ 0.6–0.8) prior to inoculation. A 5% (v/v) inoculum was introduced into each MEC: GM systems inoculated with *G. metallireducens* only, SO systems inoculated with *S. oneidensis* MR-1 only, and GM+SO systems inoculated with both strains at a 1:1 (v/v) ratio. All inoculations were performed under a sterile and anaerobic N_2_:CO_2_ (80:20) atmosphere.

### 2.2 MEC Configuration and Electrochemical Measurements

Single-chambered MECs were constructed from 125 mL serum bottles fitted with silicone/PTFE rubber septa and operated with a 100 mL working volume. Anaerobic conditions were maintained by sparging the headspace and medium with N_2_:CO_2_ (80:20, v/v) for 45 min prior to inoculation. Each MEC was assembled in a three-electrode configuration. The working electrode (bioanode) was carbon cloth (1071, Fuel Cells Etc., TX, USA) with a surface area of 6.25 cm^2^. The counter electrode was 60% Pt-coated carbon cloth (0.5 mg cm^–2^; W1S1011, Fuel Cells Etc., TX, USA), also 6.25 cm^2^. An Ag/AgCl electrode in 3 M KCl (RE-1CP, ALS, Tokyo, Japan) served as the reference. Electrical connections were made with titanium wire. All MECs were maintained at 37 °C and continuously stirred at 350 rpm to ensure homogeneous mixing.

Three bioanode configurations were operated in triplicate: *Geobacter metallireducens* (GM), *Shewanella oneidensis* MR-1 (SO), and the GM+SO coculture. The first 58 days constituted an acetate acclimation phase (1600 mg L^–1^ sodium acetate), followed by a transitional feed of 800 mg L^–1^ sodium acetate + 5 mM ethanol (EtOH) and a graded ethanol-only series of 5, 10, 15, and 20 mM, each maintained for 4 days.

The MECs were connected to a MultiEmStat3+ potentiostat (PalmSens, Netherlands) operating in three-electrode mode, with the bioanode as the working electrode, the Pt-coated carbon cloth as the counter electrode, and Ag/AgCl (3 M KCl) as the reference. The bioanode was poised at +0.3 V vs Ag/AgCl, but the current at this potential was not logged. Cyclic voltammetry (CV) was recorded from −0.8 to 0.8 V vs Ag/AgCl, and linear sweep voltammetry (LSV) from −0.5 to 0.8 V vs Ag/AgCl, both at a scan rate of 5 mV s^–1^. CV and LSV were performed daily under turnover conditions to monitor the redox behavior and oxidation current response of each bioanode across all substrate phases. Hydrogen evolution was the expected cathodic reaction; however, cathodic products, cell voltage, and Coulombic efficiency were not quantified, because the study was designed to compare bioanode performance.

### 2.3 Substrate Degradation

Acetate degradation was quantified using an HPLC system, (Dionex Ultimate 3000, Germering, Germany) with a UV/Visible detector (λ = 210 nm) along with an Agilent HPLC column (Eclipse XDB-C18, 3 μm) (Vilnius, Lithuania), with dimensions of 4.6 × 150 mm. The eluent was H_2_O: acetonitrile (CH_3_CN), 98:2 with 0.2% orthophosphoric acid (H_3_PO_4_), maintaining a pH of 2–3, a flow rate of 1 mL min^–1^, and a 25 °C column oven temperature.

Ethanol was quantified by headspace gas chromatography with flame ionization detection (HS-GC-FID), using a Thermo Scientific TRACE 1610 GC equipped with a TriPlus RSH autosampler (Thermo Fisher Scientific, USA). Samples (1 mL) were sealed in 20 mL headspace vials and equilibrated at 30 °C for 1 min with agitation (250 rpm). An 800 μL headspace aliquot was injected in split mode (16.7:1; inlet 180 °C) onto an Rtx-1 capillary column (30 m × 0.25 mm i.d, 0.25 μm; Restek, USA) with helium (He) as the carrier gas (1.0 mL min^–1^). The oven was kept at 35 °C for 3.5 min, ramped up to 95 °C at a rate 30 °C min^–1^, and held for 0.5 min (total run time 6 min). The FID was operated at 250 °C. Ethanol (retention time was 2.49 min) was quantified by external calibration against standards prepared in the same matrix. All samples were analyzed in triplicates.

### 2.4 Nuclear Magnetic Resonance (NMR) Spectroscopy

^1^H NMR spectra were recorded on a Bruker Avance III HD 400 MHz NMR Spectrometer equipped with a 5 mm BBFO probe and *Z*-axis gradient coil. The samples were dissolved in a mixture of 90% H_2_O/10% D_2_O.

### 2.5 Biofilm and Planktonic Metabolic Activity (MTT Assay)

The metabolic activity of anodic biofilms was estimated with a colorimetric MTT assay, which measures the reduction of MTT to formazan by metabolically active cells. At the end of the experiment, anodes were cut into 2 cm^2^ sections, gently rinsed with phosphate-buffered saline (PBS), and transferred to 15 mL centrifuge tubes containing 5 mL of 3-(4,5-dimethylthiazol-2-yl)-2,5-diphenyltetrazolium bromide (MTT) solution (0.5 mg mL^–1^; Merck). Samples were incubated in the dark at 37 °C for 2 h to allow metabolically active microorganisms to reduce MTT to insoluble purple formazan crystals. The MTT solution was then removed and replaced with 5 mL of a 1:1 (v/v) mixture of dimethyl sulfoxide (DMSO) and ethanol to dissolve the formazan crystals. After further incubation at 37 °C for 30 min, the absorbance of the extracted solution was measured at 540 nm using a GENESYS 10S UV–Vis Spectrophotometer (Thermo Fisher Scientific). Higher absorbance indicates greater reductive activity per anode area. It does not directly measure cell number or biomass.

Planktonic metabolic activity was determined using the same MTT assay described for biofilm samples. Briefly, 1 mL of reactor liquid was collected and centrifuged at 10,000 rpm for 10 min to concentrate suspended cells. The resulting pellet was subjected to the same MTT incubation, formazan extraction, and absorbance measurement procedures described above. Absorbance at 540 nm was used as an indicator of planktonic metabolic activity.

### 2.6 Microbial Diversity Analysis and PICRUSt2 Analysis

The analysis of the relative microbial distribution on the anodes of the GM, SO, and GM+SO MECs was performed by HyLabs Ltd. DNA was extracted using the MagCore Genomic DNA Tissue Kit (RBC Bioscience). A 16S rRNA gene library was prepared for sequencing on the Illumina platform (San Diego, CA, USA) using a two-step PCR protocol. The V4 region of the 16S rRNA gene was amplified using the forward primer CS1-515F (GTGYCAGCMGCCGCGGTAA) and the reverse primer CS2-806R (GGACTACNVGGGTWTCTAAT). Sequencing was performed on an Illumina MiSeq system using a v2-500PE kit to generate 2 × 250 bp paired-end reads. Demultiplexing was performed using CLC Genomics Workbench (QIAGEN) to obtain FASTQ files for each sample.

Bioinformatic processing was conducted in QIIME 2. Sequence quality filtering, denoising, chimera removal, and amplicon sequence variant (ASV) inference were performed using the DADA2 algorithm within QIIME 2. Representative ASVs were aligned to the SILVA rRNA database using a 99% similarity threshold to generate taxonomic assignments and construct abundance tables.

Functional potential was predicted with PICRUSt2 (Phylogenetic Investigation of Communities by Reconstruction of Unobserved States).^26^ The ASV table and representative sequences were placed onto a reference phylogeny, and gene-family abundances were inferred by hidden-state prediction, normalized to the 16S rRNA copy number, and weighted by ASV abundance. Enzyme commission (EC) numbers were mapped to MetaCyc pathways to reconstruct per-sample metabolic profiles, with the focus on enzymes involved in ethanol oxidation, acetate metabolism, the TCA cycle, and c-type cytochrome-mediated electron transfer.^27^ Functional potential was predicted from 16S rRNA gene amplicon data using PICRUSt2. Weighted nearest-sequenced-taxon index (NSTI) values were low across all samples (mean ± SD = 0.013 ± 0.004; range 0.008–0.018), well below the default quality threshold (NSTI < 2), indicating that the amplicon sequence variants were closely related to genomes in the reference database and supporting high confidence in the predicted functional profiles. PICRUSt2 predictions reflect inferred genetic potential, not gene expression or enzyme activity, and were interpreted accordingly.

### 2.7 Statistical Analysis

Each configuration (GM, SO, and GM+SO) was operated in three independent reactors (n = 3). The reactor was treated as the experimental unit, and repeated measurements from the same reactor were not treated as independent replicates. Data are reported as mean ± SD of the three reactors. Statistical analyses were conducted in Microsoft Excel and R, with significance set at *p* < 0.05. Differences among configurations, substrate concentrations/phases, and sampling times were evaluated using linear mixed-effects models, with configuration, substrate consumption (Figure 3), and time treated as fixed effects and reactor treated as a random intercept. Pairwise comparisons were performed using estimated marginal means with Tukey adjustment. For community data, Bray–Curtis dissimilarity was visualized by PCoA and tested by PERMANOVA (999 permutations); alpha diversity (Shannon index) was compared by the Kruskal–Wallis test. Exact p-values are reported throughout the analysis. Because the ethanol concentrations were applied in increasing order in the same reactors, the effect of concentration cannot be fully separated from the effects of operating time and prior exposure.

## 3 Results

### 3.1 Current Density Profile throughout the Experiment

MEC system performance was tracked over the 82-day period by performing daily linear sweep voltammetry (LSV) and recording the current density at 0.8 V vs Ag/AgCl (Figure 1). The three MEC configurations exhibited markedly different polarization profiles, with the consistent hierarchy GM+SO > GM > SO observed across all phases of operation.

**Figure 1:**
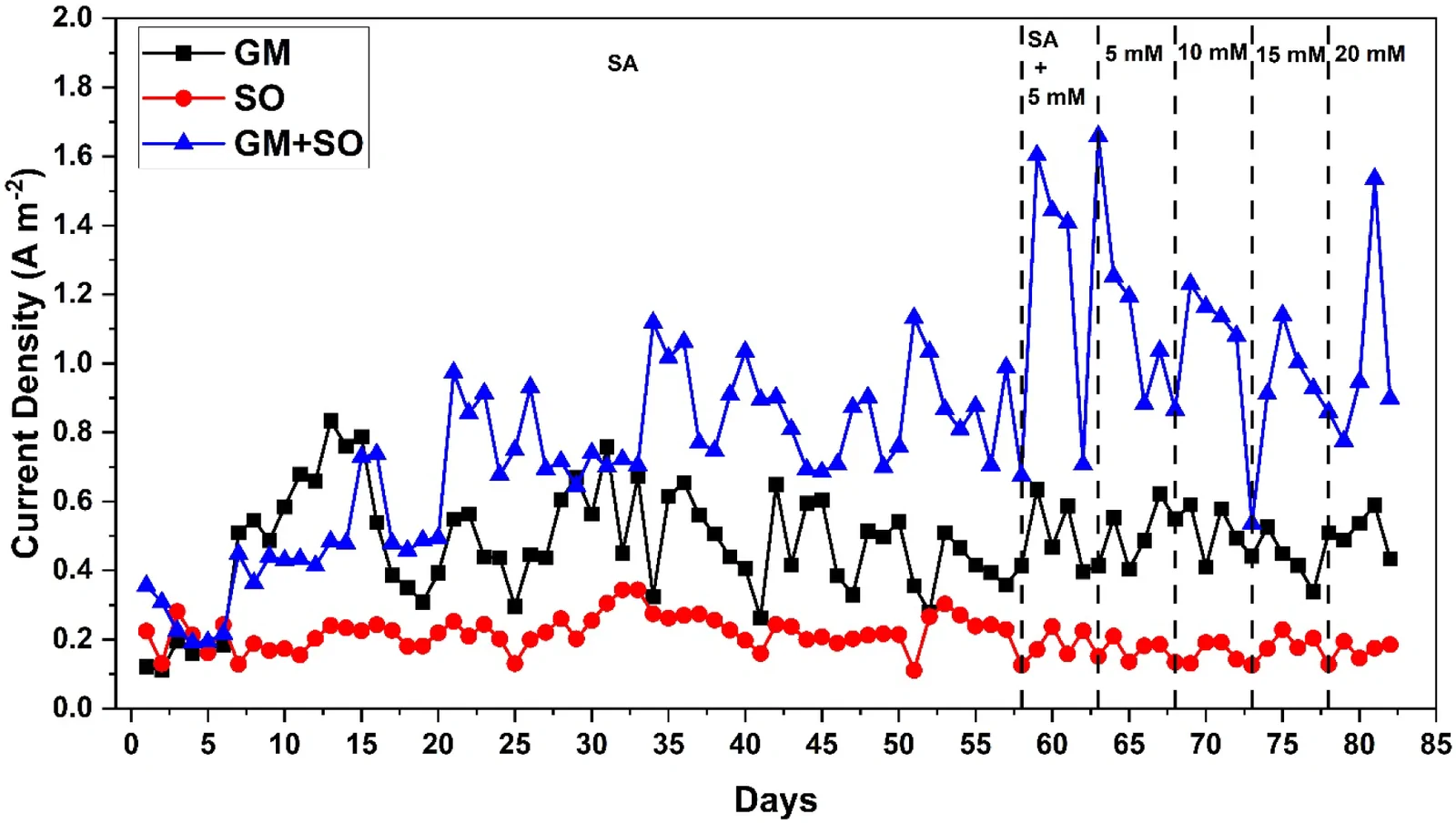
Current density profile throughout the experiment over 82 days for GM, SO, and GM+SO MEC systems. Vertical dashed lines indicate substrate-transition: 1600 mg L^–1^ sodium acetate (SA), 800 mg L^–1^ SA + 5 mM EtOH, 5 mM, 10 mM, 15 mM, and 20 mM (EtOH).

During the acetate phase when the systems were fed with 1600 mg L^–1^ sodium acetate (SA), GM+SO generated the highest current density, 1.13 A m^–2^ at day 51, followed by GM, which generated 0.83 A m^–2^ on day 13; SO generated the least among the three configurations, 0.34 A m^–2^ on day 32. The first 6 days were characterized by low current densities (<0.35 A m^–2^) and minimal differentiation among the three systems, consistent with the lag phase required for bioanode colonization and extracellular matrix establishment. After day 7, the polarization currents rose progressively as the electroactive biofilms matured. Throughout the acetate phase, the measurements reflected periodic substrate-replenishment cycles, in which bioanodes transiently approached substrate limitation between feeds before recovering upon medium replacement. Following substrate transition on day 58 to a transitional feed of 800 mg L^–1^ sodium acetate and 5 mM ethanol, the GM+SO coculture displayed an immediate and pronounced surge in polarization current density, reaching 1.60 A m^–2^ by day 59; this was a 2.7-fold enhancement over GM, which was 0.60 A m^–2^, and a 9.4-fold enhancement over SO, which was 0.17 A m^–2^. The rapid response indicated that the pre-established GM+SO biofilm, which had matured during the preceding acetate phase, was metabolically primed to engage with the new carbon source without requiring substantial community restructuring. Across the subsequent ethanol-only phases from days 63–82 (5 mM, 10 mM, 15 mM, and 20 mM), GM+SO maintained peak polarization current densities of 1.66, 1.23, 1.14, and 1.54 A m^–2^, respectively. GM monocultures achieved between 0.33 and 0.62 A m^–2^, while SO monocultures generated current densities consistently below 0.21 A m^–2^ across all ethanol concentrations, with the thin anodic biofilms and weak electrode coupling reported for *S. oneidensis*. Current density in GM+SO did not increase in proportion to ethanol concentration (5–20 mM), suggesting that substrate supply did not limit current over this range; the limiting step was not identified.

Overall, the reproducibility of this performance ranking across both substrate phases provided an initial line of evidence for synergistic cooperation between *G. metallireducens* and *S. oneidensis* within the bioanode community. The coculture brings together an organism known for direct electron transfer (DET) via outer-surface cytochromes (*Geobacter*) and one that relies largely on flavin-mediated electron transfer (MET) (*Shewanella*), although the relative contribution of each route was not resolved here. *S. oneidensis* is capable of oxidizing ethanol to acetate.^28^ Previous studies have demonstrated that *S. oneidensis* can establish mutualistic interactions with *E. coli* through the secretion of flavins, which act as extracellular electron shuttles and facilitate electron transfer to electrodes. Such syntrophic interaction suggests that *S. oneidensis* may enhance electron flux within mixed microbial communities, thereby improving the electrochemical performance of bioelectrochemical systems.^29^ *S. oneidensis* is known to secrete flavins that can act as electron shuttles,^30,31^ but flavins were not measured here, and their contribution in the present system remains unknown could, in principle, complement the cytochrome-based electron transfer of *G. metallireducens*. Whether such an interaction operates in the present system was not tested. One possible explanation is sequential substrate conversion, in which *S. oneidensis* oxidizes ethanol to acetate that *G. metallireducens* then consumes. However, because acetate supplementation alone did not raise GM current to coculture levels, additional interactions are likely to contribute.

### 3.2 Electrochemical Anode Characterizations

#### 3.2.1 Cyclic Voltammetry

Cyclic voltammograms acquired under turnover conditions at each substrate phase (Figure 2) provided mechanistic insight into electron-transfer behavior. Under acetate (1600 mg L^–1^) (Figure 2a), GM exhibited a single broad anodic feature with an oxidation onset potential near −0.15 V and an anodic peak potential at +0.23 V (0.38 A m^–2^); no corresponding cathodic peak was observed, indicating an irreversible oxidation reaction. SO produced an essentially featureless, capacitive response, consistent with negligible faradaic turnover. The coculture GM+SO exhibited a markedly larger response, with an anodic onset potential near +0.50 V reaching 1.36 A m^–2^, and a cathodic peak potential at +0.43 V, more than 3.5-fold higher than in the GM.

**Figure 2:**
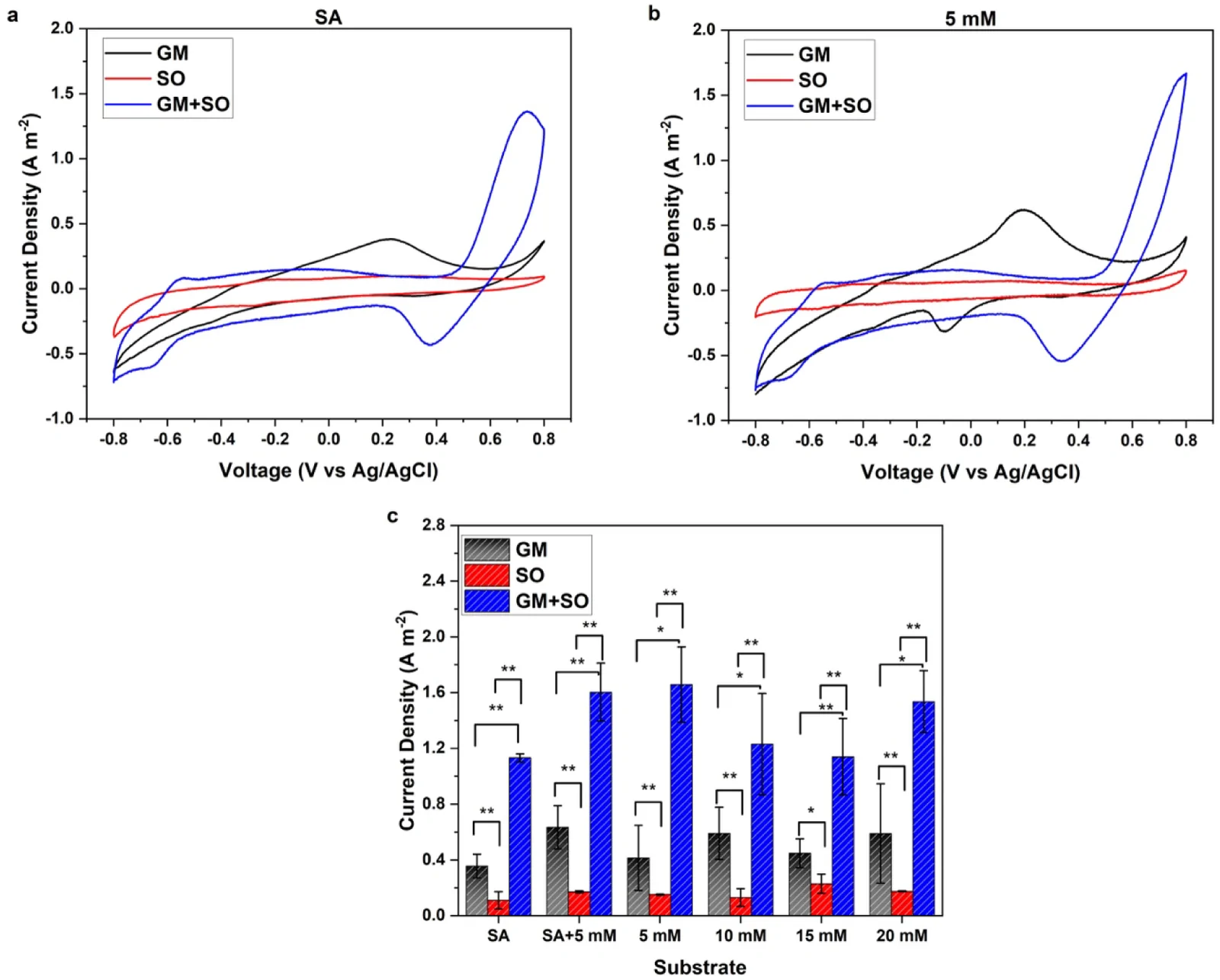
Cyclic voltammograms (5 mV s^–1^, −0.8 to 0.8 V vs Ag/AgCl) of GM, SO, and GM+SO MECs operation under each substrate condition: (a) SA, (b) 5 mM ethanol, (c) LSV current densities at 0.8 V vs Ag/AgCl. GM+SO significantly exceeded both GM and SO (*p* ≤ 0.01** at SA, SA + 5 mM, 5 mM; *p* ≤ 0.05* at 10 mM, 20 mM; *p* ≤ 0.01** at 15 mM).

With 5 mM ethanol (Figure 2b), GM exhibited a more defined and quasi-reversible reaction, comprising an anodic peak potential at +0.20 V (0.62 A m^–2^) and a cathodic peak at −0.10 V. SO again remained featureless, while the coculture GM+SO showed a steep catalytic anodic wave (onset potential +0.47 V, 1.66 A m^–2^), together with a distinct cathodic peak at +0.33 V. In every case, the anodic peak current density of the coculture greatly exceeded that of either pure culture, indicating a synergistic enhancement of electron transfer. SO produced substantially lower current densities over the entire potential window, with a relatively featureless and compressed voltammogram. The weak response suggested limited direct electron transfer and lower catalytic activity under the tested conditions, likely reflecting the SO dependence on soluble flavin mediators rather than extensive electrode colonization.^30^ This suggests that additional redox-active components are present in the GM+SO biofilm. These may include outer-membrane cytochromes and secreted flavins, but they cannot be identified from the present voltammograms.

Across the 5–20 mM ethanol range, GM+SO retained well-defined catalytic waves with only a modest decrease at 20 mM, indicating preserved redox connectivity even at increased substrate loadings. The reversibility of the GM+SO redox response, with closely spaced anodic and cathodic features and a small peak-to-peak separation, indicates quasi-reversible redox behavior. Together, these observations show that the coculture biofilm has a larger and more redox-active electrochemical interface than either monoculture.

The electrocatalytic activity of each inoculum across carbon sources was studied using LSV analysis. The peak current densities were measured at +0.8 V (vs Ag/AgCl) (Figure 2c). *S. oneidensis* (SO) produced the lowest current densities across all tested substrates, below 0.21 A m^–2^, with no consistent dependence on carbon source identity. *G. metallireducens* (GM) yielded intermediate values of 0.36 ± 0.09 to 0.63 ± 0.15 A m^–2^, depending on substrate. The coculture (GM+SO) generated substantially higher current densities under all conditions: sodium acetate alone supported 1.13 ± 0.03 A m^–2^, representing a 1.8 to 3.1-fold increase over GM, and a 4.9 to 10.3-fold increase over SO under equivalent substrate conditions. Ethanol supplementation further elevated coculture current density in a concentration-dependent manner. The highest values were recorded in 5 mM ethanol, 1.66 ± 0.27 A m^–2^; and in the SA + 5 mM ethanol mixture, 1.60 ± 0.21 A m^–2^. At 10 and 15 mM ethanol, current density declined to 1.23 ± 0.36 and 1.14 ± 0.27 A m^–2^, respectively, before recovering to 1.54 ± 0.22 A m^–2^ at 20 mM. These data indicated that ethanol served as an effective electron donor for the coculture, with peak electrocatalytic activity at 5 mM, and suggested partial inhibition or metabolic rerouting at intermediate concentrations (10–15 mM). As shown in Table 1, LSV current from the GM+SO coculture exceeded the sum of the two monocultures currents across every substrate concentration (1.7- to 2.9-fold higher), with the largest synergistic gain at 5 mM ethanol alone (2.9-fold) and the smallest at 15 mM ethanol (1.7-fold). The coculture current density exceeded the arithmetic sum of the monoculture means across the tested substrate conditions, supporting an enhancement beyond the additive expectation.

**Table 1:** LSV Currents Sum of GM and SO Compared to GM+SO

Substrate	Sum of GM and SO (A m^–2^)	GM+SO (A m^–2^)
SA (1600 mg L^–1^)	0.46 ± 0.14	1.13 ± 0.02
SA (800 mg L^–1^) + 5 mM EtOH	0.80 ± 0.16	1.60 ± 0.20
5 mM EtOH	0.56 ± 0.23	1.66 ± 0.27
10 mM EtOH	0.72 ± 0.25	1.23 ± 0.36
15 mM EtOH	0.67 ± 0.17	1.14 ± 0.27
20 mM EtOH	0.76 ± 0.35	1.54 ± 0.22

The pronounced enhancement of the coculture was consistent with the complementary electron transfer machinery of the coculture. *G. metallireducens* transfers electrons predominantly through outer-membrane c-type cytochromes (direct electron transfer), whereas *S. oneidensis* is well-known to secrete flavins (riboflavin and flavin mononucleotides) that function both as soluble redox shuttles and as bound cofactors of the Mtr/Omc cytochromes, enabling mediated electron transfer.^12,30^ The catalytic anodic wave and cathodic peaks recorded for the coculture therefore could not be assigned to a discrete flavin couple. Rather, the enhanced current density may reflect a combination of cytochrome-based electron transfer by GM, flavin-mediated transfer by SO, and metabolic cross-feeding, but the present data do not separate these contributions in which ethanol fermentation products generated by SO are further oxidized by GM.

Most prior coculture work in bioelectrochemical systems has used *G. sulfurreducens* rather than *G. metallireducens*, and almost all of it has been run on acetate or lactate, on which neither partner is kinetically limited.^5^ In previous coculture studies, *S. oneidensis* monocultures generated the lowest electrochemical response and power density, 88 ± 8 mW m^–2^, whereas cocultures exhibited the highest cyclic voltammetry peak currents and achieved power densities of 195 ± 8 mW m^–2^; demonstrating that synergistic interactions can significantly increase current generation and extracellular electron transfer.^23^ *S. oneidensis* MR-1 was unable to directly utilize glycerol and produced negligible voltage (<3 mV) when grown alone, while cocultivation with *Klebsiella pneumoniae* J2B nearly doubled the current density (12.3 vs 6.8 mA m^–2^), increased the cyclic voltammetry oxidation peak from 0.3 to 0.5 mA at −0.35 V, and raised the maximum power density from 1.84 to 2.15 mW m^–2^. These results demonstrated that *S. oneidensis* can substantially enhance extracellular electron transfer through syntrophic cooperation and complementary electron-transfer pathways, even when it cannot directly metabolize the primary substrate.^24^

Extracellular electron transfer is associated with significant potential losses, representing electron-derived energy dissipated through activation overpotentials, metabolic resistance, mediator diffusion limitations, and inefficient electron-transfer kinetics. DET generally exhibits higher energetic losses than MET, due to cytochrome orientation, electron tunneling distance, and biofilm conductivity constraints.^32^ In previous coculture studies, *S. oneidensis* produced only transient and comparatively low current densities of 0.034 ± 0.011 mA cm^–2^, nearly an order of magnitude lower than those generated by *G. sulfurreducens* (0.39 ± 0.09 mA cm^–2^), with current rapidly declining to near-zero despite substantial lactate consumption and acetate accumulation. This indicated that *S. oneidensis* contributes primarily through substrate conversion and flavin-mediated electron shuttling, rather than sustained direct anodic respiration.^18^

### 3.3 Substrate Utilization

Sodium acetate was chosen as the initial substrate because *G. metallireducens* can oxidize it directly without a fermentation step, giving a clean, literature-comparable baseline of each configuration’s intrinsic electrogenic capacity. Ethanol was then introduced, first as a cosubstrate with acetate, then as the sole carbon source at increasing concentrations (5–20 mM). The purpose was to test performance on a substrate representative of alcohol-rich industrial effluents, the target application for this consortium. Although *G. metallireducens* can oxidize ethanol directly, it does so with low efficiency, so this substrate specifically probed whether *S. oneidensis‘s* oxidative capacity could functionally complement GM’s electron transfer pathway. The concentration escalation tested the robustness of that cooperation, including any onset of substrate inhibition.

#### 3.3.1 Acetate

HPLC quantification of acetate removal (Figure 3a–b) showed that all three MEC configurations consumed acetate efficiently, but with distinct kinetic profiles. With sodium acetate on day 36 (SA 48 h) and day 38 (SA 96 h) after freshly added media, SO achieved the fastest early removal, reaching 76% within 48 h, followed by GM+SO at 68% and GM at 50% (Figure 3a). After 96 h, all configurations had reached near-complete removal, with GM+SO bringing acetate removal to essentially 100%, ahead of SO (99.8%) and GM (99%) (Figure 3b). When acetate was cosupplied with 5 mM ethanol (800 mg L^–1^ acetate), the same ranking held at 48 h: SO removed 69%, GM+SO 59%, and GM 38%; up to 96 h, GM+SO achieved 99.8% removal, followed by SO at 99% and GM at 94%. The slower acetate consumption by GM in the presence of ethanol suggested competitive flux through the central acetyl-CoA node. In contrast, GM+SO appeared to process both substrates concurrently without comparable interference, so that acetate (both the externally supplied form and that generated as an ethanol-oxidation intermediate) was rapidly turned over by the consortium. Although SO removed acetate fastest, it produced the least current, so most electrons from the acetate it removed were not recovered at the anode. Because *S. oneidensis* MR-1 is generally reported to oxidize acetate poorly under anaerobic conditions, noninoculated populations (Section 3.5) may have contributed to this removal. Taken together, GM+SO combined rapid acetate removal with the highest current density.

**Figure 3:**
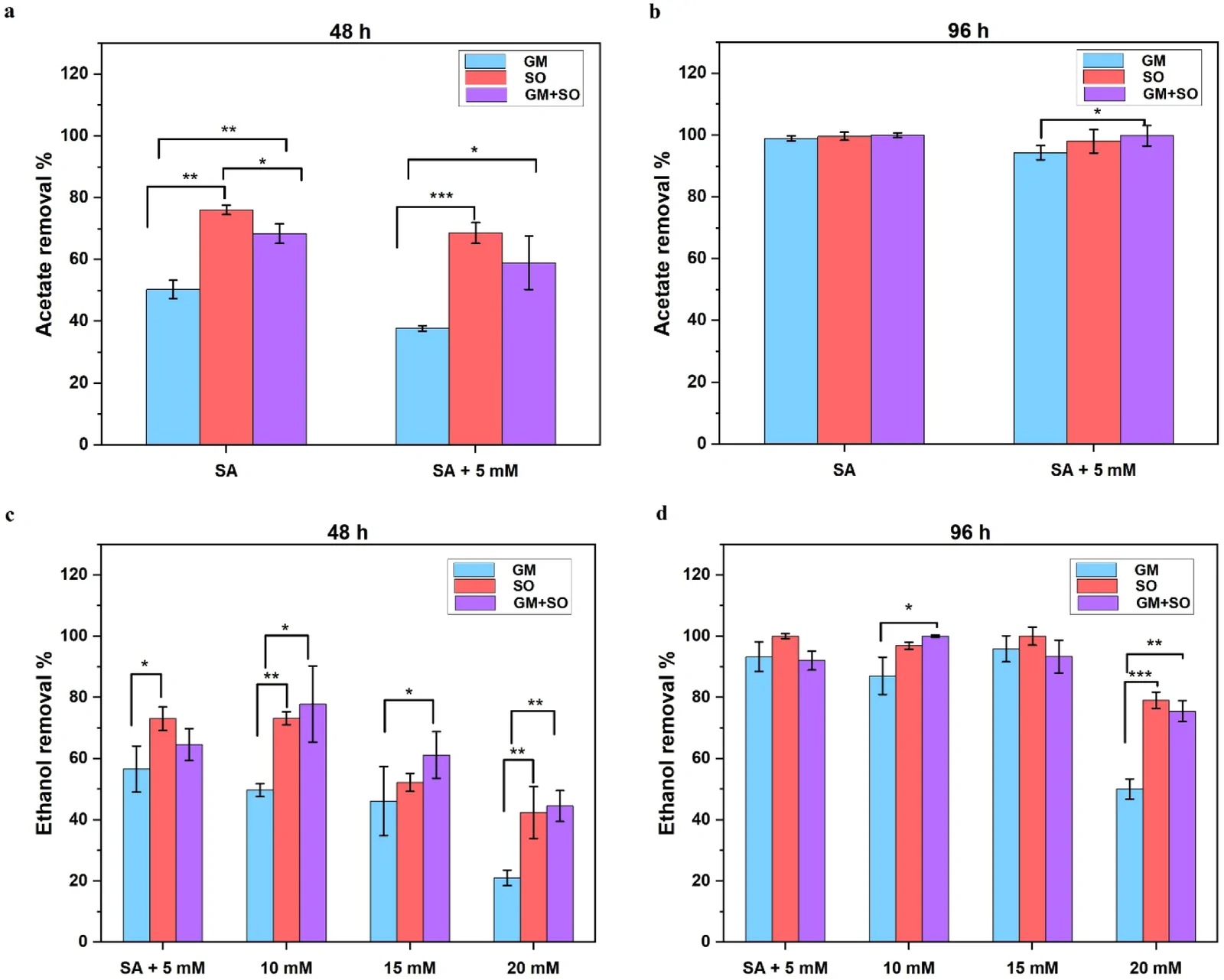
Sodium acetate and ethanol degradation in GM, SO, and GM+SO MECs. (a) Sodium acetate 48 h, (b) Sodium acetate 96 h, (c) Ethanol 48 h and (d) Ethanol 96 h. Values are means ± SD of three independent reactors. Differences between configurations were tested with a linear mixed-effects model (configuration × concentration × time, reactor as random effect), followed by Tukey-adjusted pairwise comparisons within each concentration and time point. *p ≤ 0.05, **p ≤ 0.01, ***p ≤ 0.001.

#### 3.3.2 Ethanol Degradation

Gas chromatographic analysis of ethanol consumption (Figure 3c–d) was quantified by GC-FID using an external standard calibration curve, showing degradation across all MECs. This is an established approach for volatile alcohols, as ethanol partitions readily into the GC system by either direct liquid injection or headspace sampling.

When 5 mM ethanol was cosupplied with acetate (SA + 5 mM), ethanol removal at 48 h was partial: 73% for SO, 64% for GM+SO, and 56% for GM. It approached completion by 96 h; SO 100%, GM 93%, GM+SO 92%. The slower early removal reflected the simultaneous presence of acetate. When 5 mM ethanol was supplied as the sole substrate, all three configurations removed the entire substrate within 48 h, indicating that this loading was below the catalytic ceiling of even the SO monoculture. At higher ethanol-only loadings, the coculture advantage became clearer at 48 h. At 10 mM, GM+SO achieved the most rapid removal, 78%, ahead of SO, 73%, and GM, 50%; and by 96 h GM+SO had reduced ethanol to nondetectable levels, while GM removed 87%. At 15 mM, GM+SO removed 61% at 48 h, comparable to SO at 52% and slightly above GM at 46%; by 96 h all three configurations had cleared ≥93% of the substrate.

At the highest loading of 20 mM, removal at 48 h was lower for all configurations: GM+SO, 44%, SO, 42%, and GM, 21%. By 96 h a clear divergence emerged: GM removed only 50%, whereas SO and GM+SO removed robust kinetics at 79% and 75%, respectively. The decline in GM performance at 20 mM was consistent with substrate inhibition, while the continuing performance of the GM+SO and SO MECs indicated that *Shewanella* contributed meaningful kinetic capacity at high ethanol loadings. The latter is likely via NAD^+^-dependent alcohol dehydrogenase activity (EC 1.1.1.1), which operates independently of c-type cytochrome saturation. Although SO alone degraded ethanol at rates comparable to the coculture, its low current production demonstrated that substrate consumption alone does not guarantee effective extracellular electron transfer. The superior electrochemical performance of GM+SO was associated with higher LSV current density despite similar or comparable substrate removal, indicating that substrate consumption alone does not predict anodic current generation. GM showed reduced ethanol removal at 20 mM, although the contribution of substrate inhibition cannot be separated from cumulative exposure and biofilm aging because the concentrations were applied sequentially.

Substrate degradation rates tracked over 48 h (Table 2) paralleled the removal trends, with GM+SO sustaining the highest or near-highest acetate and ethanol degradation rates across most loadings, while GM exhibited pronounced rate suppression at elevated ethanol concentrations (≥20 mM), consistent with substrate inhibition. These kinetic data, combined with the coculture’s superior current output, supported a mechanistic interpretation in which enhanced catabolic flux is efficiently coupled to extracellular electron transfer, rather than the two processes operating independently.

**Table 2:** Initial Substrate Degradation Rate for 48 h^a^

Substrate	Concentration	GM	SO	GM+SO
Acetate (mg d^–1^)	Acetate (1600 mg L^–1^)	402 ± 23	608 ± 12**	547 ± 25**
	SA + 5 mM (800 mg L^–1^)	142 ± 3	274 ± 13**	235 ± 34*
Ethanol (mM d^–1^)	SA + 5 mM L^–1^	1.45 ± 0.19	1.86 ± 0.10*	1.60 ± 0.45
	5 mM L^–1^	2.5 ± 0.01	2.5 ± 0.01	2.5 ± 0.01
	10 mM L^–1^	2.55 ± 1.1	3.57 ± 0.10	3.72 ± 0.55
	15 mM L^–1^	3.46 ± 0.80	3.71 ± 0.20	4.68 ± 0.55
	20 mM L^–1^	2.05 ± 0.25	4.14 ± 0.80*	4.43 ± 0.50**

a Values are means ± SD of three independent reactors. Asterisks denote Tukey-adjusted differences from GM at the same concentration, based on a linear mixed-effects model (Section 2.7): *p < 0.05, **p < 0.01. Rates were calculated over the first 48 h of each feeding cycle.

When glycerol was provided as the sole carbon source, *S. oneidensis* consumed 6.5 mM glycerol and accumulated 5.9 ± 0.1 mM acetate in monoculture. In contrast, acetate was not detected in coculture with *G. sulfurreducens*, demonstrating that acetate produced by *S. oneidensis* was rapidly utilized by *G. sulfurreducens*. This metabolic cross-feeding illustrated syntrophic cooperation, in which one partner converts the primary substrate into an intermediate that is subsequently consumed by the second organism to support enhanced extracellular electron transfer and current generation.^25^ Previously, studies used a coculture of *Klebsiella pneumoniae* J2B in fermented glycerol to produce a mixture of reduced and oxidized intermediates, where *S. oneidensis* MR-1 selectively consumed partner-derived metabolites, decreasing lactate concentrations from 7.1 ± 0.2 to 0.6 ± 0.0 mM and acetate concentrations from 5.0 ± 0.6 to 1.8 ± 0.1 mM. These changes provided direct evidence of metabolic cross-feeding and syntrophic cooperation, in which *S. oneidensis* scavenged fermentation products generated by *K. pneumoniae* J2B, thereby coupling substrate conversion to enhanced extracellular electron transfer and current generation.^24^

In a defined coculture of *S. oneidensis* with *G. sulfurreducens* and *G. metallireducens*, *S. oneidensis* consumed 7.3 mM lactate, nearly identical to the ∼8 mM consumed in monoculture, while acetate accumulated to only 5.6 mM, compared with the 6.6 mM expected from monoculture stoichiometry. This indicated that approximately 1 mM acetate produced by *S. oneidensis* was subsequently consumed by the *Geobacter* partners, providing direct evidence of syntrophic metabolic cross-feeding.^21^ Previous studies have established *G. metallireducens* as an efficient ethanol-oxidizing bacterium, consuming approximately 5 mM ethanol within the first 7 days of a defined coculture with *G. sulfurreducens* (∼0.71 mM d^–1^).^33^ In a previously reported syntrophic coculture with *Methanosarcina thermoacetophila*, *G. metallireducens* completely oxidized 20 mM ethanol within 15 days, corresponding to an average ethanol consumption rate of approximately 1.33 mM d^–1^. Acetate accumulated transiently to ∼3.5 mM by day 7 and subsequently declined to near zero, confirming that *G. metallireducens* first converted ethanol to acetate, which was then consumed by the methanogenic partner. This provided direct quantitative evidence for the robust ethanol-oxidizing capacity of *G. metallireducens* and its role in syntrophic metabolic cross-feeding.^34^

#### 3.3.3 ^1^H NMR Confirmation of Acetate Formation

To verify that ethanol oxidation proceeded through acetate, ^1^H NMR spectra were acquired from samples collected after 48 h of incubation under ethanol-only feeding at an initial concentration of 10 mM (Figure S1). The diagnostic acetate methyl singlet at δ 1.83–1.84 ppm was clearly observed in GM, SO, and GM+SO. The complementary quartet pattern of unreacted ethanol (CH_3_ at δ 1.08–1.12 ppm; CH_2_ at δ 3.55–3.60 ppm) decreased over time in all systems, in agreement with the GC degradation data. The NMR spectra confirmed the presence of acetate as a soluble intermediate during ethanol consumption in GM, SO, and GM+SO. The presence of additional SO-specific peaks suggested that *S. oneidensis* performs a more diversified, fermentative metabolism, including potential lactate or formate production, rather than the clean two-step oxidation observed in *Geobacter*-rich biofilms. Acetate formation profiles measured by HPLC (Figure S2) showed transient peaks at 48 h for 15 mM (23.8 ± 8.4 ppm) and 20 mM ethanol (16.8 ± 16.8 ppm) in GM+SO. These observations are consistent with acetate formation as an intermediate during ethanol oxidation; however, downstream oxidation of acetate to CO_2_ was not directly measured. Acetate transfer may therefore contribute to, but does not fully explain, the higher current of the coculture.

*S. oneidensis* MR-1 can utilize CO_2_ for acetate production in microbial electrosynthesis systems via electroactive metabolism and reductive carbon fixation pathways.^35^ Our quantitative evidence closely paralleled our ethanol-fed consortium, in which *S. oneidensis* oxidized ethanol to acetate and *G. metallireducens* subsequently consumed the acetate, consistent with possible metabolic cooperation between the organisms that enhanced extracellular electron transfer and current production.^21^

### 3.4 Biofilm and Planktonic Metabolic Activity in MECs (MTT Assay)

Quantitative MTT assay of harvested biofilms showed that biofilm metabolic activity followed a similar pattern to current generation for SO relative to the other configurations (Figure 4). The OD_540 nm_ values for GM (3.22 ± 0.09) and GM+SO (3.17 ± 0.16) were statistically indistinguishable (Tukey HSD, *p* > 0.5) and approximately 2.3-fold higher than SO (1.42 ± 0.33; *p* < 0.001). Planktonic metabolic activity was similar in all three reactors (3.04–3.29), indicating that the lower biofilm activity of SO was not due to lower activity in the bulk. The lower biofilm MTT signal in SO indicates lower metabolic activity per sampled anode area under the tested conditions. This does not by itself establish differences in biomass or biofilm thickness. One possibility is that the *Geobacter*-rich biofilm provides a matrix that retains *S. oneidensis* cells near the electrode, although this was not examined directly.

**Figure 4:**
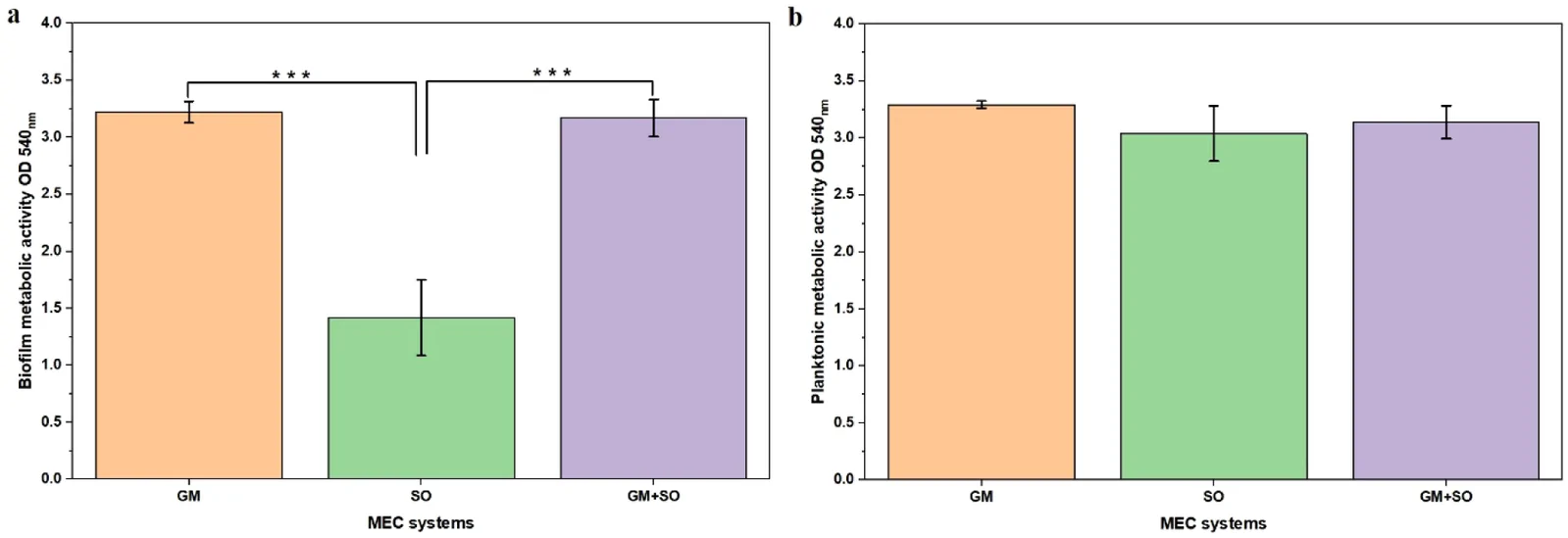
(a) Biofilm metabolic activity (OD_540 nm_) of GM, SO, and GM+SO bioanodes determined by MTT assay after 82 days of MEC operation. (b) Planktonic metabolic activity.

*Geobacter*-*Shewanella* cocultures have demonstrated that *S. oneidensis* occurs at much lower abundance, especially in electrode biofilms, consistent with its weaker surface attachment reported in previous coculture studies.^18^ Electrochemical contributions of *S. oneidensis* in coculture are consistent with its tendency to form thinner, less conductive biofilms and to rely on flavin-mediated electron transfer, whereas *Geobacter* species establish thicker, highly conductive biofilms that support sustained direct electron transfer to the anode.^36^ In previous studies electroactive biomass showed a direct correlation with maximum current output, consistent with previously reported findings.^18^

### 3.5 Microbial Community Analysis

16S rRNA amplicon sequencing of the harvested bioanodes confirmed that each MEC retained its inoculated species as the dominant community member, but also revealed clear differences in community structure, diversity, and supporting taxa between the three configurations (Figure 5).

**Figure 5:**
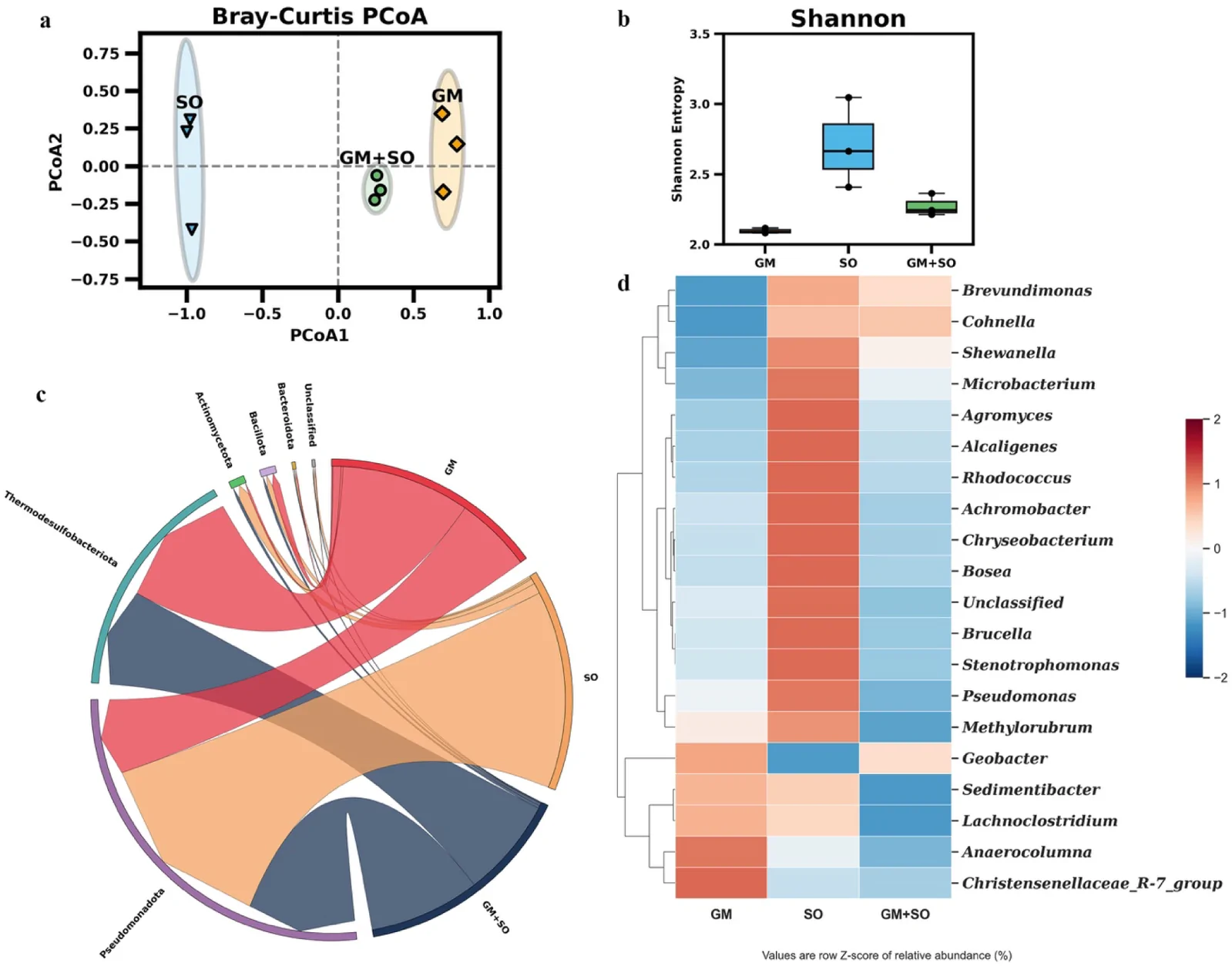
Microbial community diversity and taxonomic composition of anodic biofilms. (a) Bray–Curtis dissimilarity-based principal coordinates analysis (PCoA). (b) Shannon entropy indicating significant differences in alpha diversity. (c) Chord diagram illustrating the distribution of dominant bacterial phyla. (d) Heatmap with hierarchical clustering of the 20 most abundant genera; values are row-wise Z-scores of relative abundances.

Bray–Curtis PCoA (Figure 5a) separated the three bioanode communities into well-resolved clusters along PCoA1, with GM and SO at opposite extremes and GM+SO occupying an intermediate position closer to GM. PERMANOVA confirmed that the three communities were significantly different (pseudo-F = 22.85, p = 0.006, 999 permutations), and the tight within-group clustering of triplicates indicated good reproducibility across replicates.

At the phylum level (Figure 5c), Thermodesulfobacteriota accounted for 59.27% of reads in GM and 45.68% in GM+SO but was essentially absent in SO. Pseudomonadota dominated SO at about 91.10%, reflecting the *Shewanella* inoculum and its associated *Pseudomonas* and *Alcaligenes* populations; it remained substantial in GM+SO at about 51.15%. Bacillota and Bacteroidota were minor components across all three configurations, about <5% each, and unclassified reads stayed below 1%.

At the genus level (Figure 5d), *Geobacter* was the dominant genus in GM, observed as 59.04%, and in GM+SO was 45.45% but absent from SO. *Shewanella* was effectively absent from GM, abundant in SO at 33.95%, and retained at 19.85% in the coculture; At the end of operation, the GM+SO biofilm therefore retained 77% of the *Geobacter* relative abundance found in GM and 58% of the *Shewanella* abundance found in SO. Both inoculated species were present, although the end point data cannot show how their proportions changed over time. The reactors were inoculated aseptically but were not maintained as axenic systems over 82 days of sampling and medium replacement, and accompanying populations developed. *Alcaligenes* was abundant in all three configurations; because its abundance was comparable across reactors, it is unlikely to explain the differences between them, but as *Alcaligenes* and *Pseudomonas* are electrochemically active, their contribution to ethanol removal and current cannot be excluded. Several auxiliary genera were observed specifically in SO (*Pseudomonas*, *Achromobacter*, *Brucella*, *Rhodococcus*, *Stenotrophomonas*, *Chryseobacterium*, *Methylorubrum*), whereas a distinct cluster of genera (*Sedimentibacter*, *Lachnoclostridium*, *Anaerocolumna*, *Christensenellaceae R-7 group*) was enriched in GM and the coculture, consistent with the more reducing, anaerobic microenvironment maintained by *Geobacter*-dominated biofilms.

The alpha diversity analysis (Figure 5b) revealed a counterintuitive but mechanistically meaningful pattern. Shannon entropy was lowest in GM, at 2.08 ± 0.02; highest in SO, 2.70 ± 0.13; and intermediate in GM+SO, about 2.25 ± 0.05; with the ordering GM < GM+SO < SO confirmed by Kruskal–Wallis testing (H = 7.20, p = 0.0273) and significant in all pairwise comparisons (p = 0.0495 for each contrast). The high Shannon value of SO reflected the fact that *Shewanella*, despite being the dominant member, did not numerically overwhelm its supporting community, leaving room for a more even distribution across multiple genera. By contrast, the low Shannon value of GM was the signature of a *Geobacter*-dominated biofilm in which one taxon accounts for most reads. It is a structure that is functionally desirable for anodic respiration, even though it scores low on classical diversity metrics. The intermediate position of GM+SO reflected the coexistence of two dominant electroactive species, and The intermediate Shannon value of GM+SO is consistent with a community containing two abundant inoculated taxa.

*G. metallireducens* is one of the model organisms for direct interspecies electron transfer (DIET). Early coculture studies with *G. sulfurreducens* demonstrated that *G. metallireducens* oxidized ethanol while transferring electrons directly through conductive aggregates composed of c-type cytochromes and electrically conductive type IV pili (e-pili).^37^ In mixed-community biofilms, *S. oneidensis* MR-1 declined from 26.3% to only 1.5% of the community over two months; yet the coculture maintained superior power generation, indicating that *S. oneidensis* can exert a disproportionate functional impact despite low abundance, likely through substrate conversion and flavin-mediated electron transfer.^23^ *Alcaligenes faecalis* is a facultative anaerobic electroactive bacterium with versatile carbon and nitrogen metabolism, capable of ammonium oxidation, nitrogen fixation, and denitrification. Previous studies suggested that *A. faecalis* primarily performs extracellular electron transfer through indirect electron transfer mechanisms involving soluble mediators, while conductive materials such as carbon-black nanoparticles can further enhance mediator-assisted electron transfer efficiency and current generation in microbial electrochemical systems.^11^ *Pseudomonas aeruginosa* is a naturally electroactive bacterium that predominantly performs extracellular electron transfer through mediated electron transfer (MET) pathways involving self-produced redox-active compounds such as phenazines, enabling efficient electron shuttling between planktonic cells and the electrode.^38^

### 3.6 PICRUSt2 Analysis

PICRUSt2-predicted MetaCyc pathway abundances and EC enzyme distributions (Figure 6) were broadly consistent with the observed metabolic and electrochemical behavior. NSTI values were lowest in the coculture GM+SO: 0.010 ± 0.003 relative to either monoculture GM: 0.016 ± 0.003 and SO: 0.014 ± 0.002, consistent with a more phylogenetically well-characterized community composition in the coculture condition. GM+SO biofilms displayed the highest predicted abundance of aerobic respiration I (cytochrome c) (113,454 ± 4,725), TCA cycle V (49,376 ± 2,063), TCA cycle I (49,946 ± 2,063), and reductive TCA cycle I (45,680 ± 2,700), pathways involved in acetate oxidation to CO_2_ through the TCA cycle. The methanogenesis-from-acetate pathway was strongly represented in GM (28,491) and GM+SO (25,591) but completely absent from SO (0), confirming that the carrier capability for syntrophic acetate metabolism was associated with *Geobacter*-containing biofilms. Pyruvate fermentation to propanoate I (34,498 in GM; 31,947 in GM+SO; 6,909 in SO) was similarly enriched, providing a complementary route for carbon flux.

**Figure 6:**
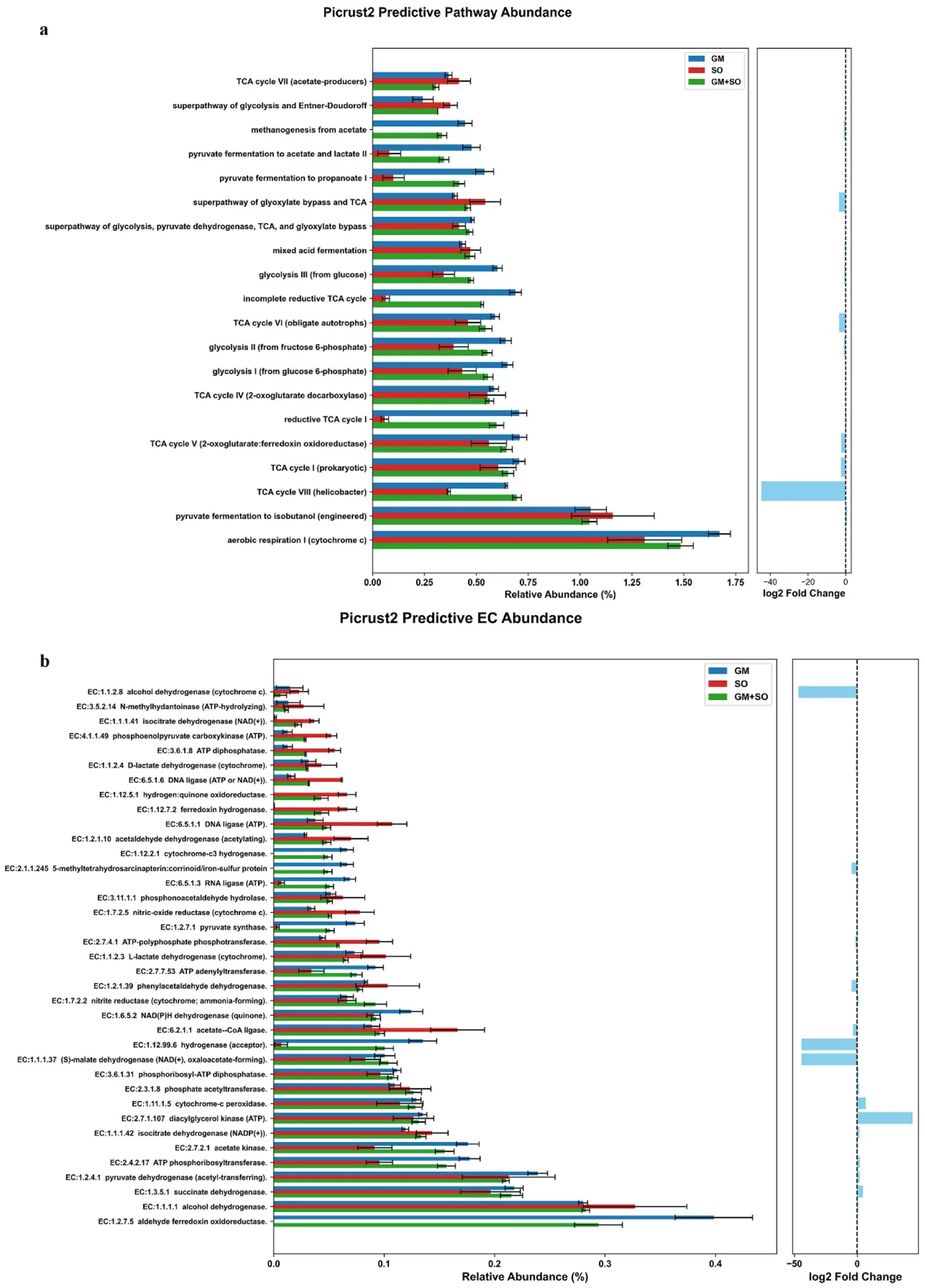
(a) PICRUSt2-predicted MetaCyc pathway abundance. (b) PICRUSt2-predicted EC enzyme abundance.

At the EC level of abundance, the most enriched ethanol-relevant enzymes in GM+SO included alcohol dehydrogenase (EC 1.1.1.1; 99,380 ± 1,223), aldehyde ferredoxin oxidoreductase (EC 1.2.7.5; 103,436 ± 7,626), acetaldehyde dehydrogenase (acetylating; EC 1.2.1.10; 16,950 ± 1,271), acetate kinase (EC 2.7.2.1; 54,487 ± 2,967), and phosphate acetyltransferase (EC 2.3.1.8; 44,549 ± 2,513). Together, these predicted enzymes would be sufficient for the hypothesized pathway as shown in Figure 7: in which ethanol is oxidized via acetaldehyde and acetate to acetyl-CoA and then through the TCA cycle to CO_2_.^15^ Of these steps, only ethanol consumption and acetate formation were measured. Crucially, cytochrome-c3 hydrogenase (EC 1.12.2.1) and cytochrome-c peroxidase (EC 1.11.1.5), both diagnostic of *Geobacter*-mediated extracellular electron transfer, were strongly represented in GM,^21^ 200 and 41,292) and GM+SO,^17^ 279 and 45,124) but were absent and lower,^35^ 333) in SO and GM+SO, respectively. Conversely, ferredoxin hydrogenase (EC 1.12.7.2) and hydrogen:quinone oxidoreductase (EC 1.12.5.1) were enriched in SO (20,673 and 20,553), consistent with its alternative respiratory chain. These predictions suggest that the coculture biofilm has the genetic potential for both ethanol activation and cytochrome-based electron transfer. Because the predictions are made at the community level, they cannot show which organism carries out each step.

**Figure 7:**
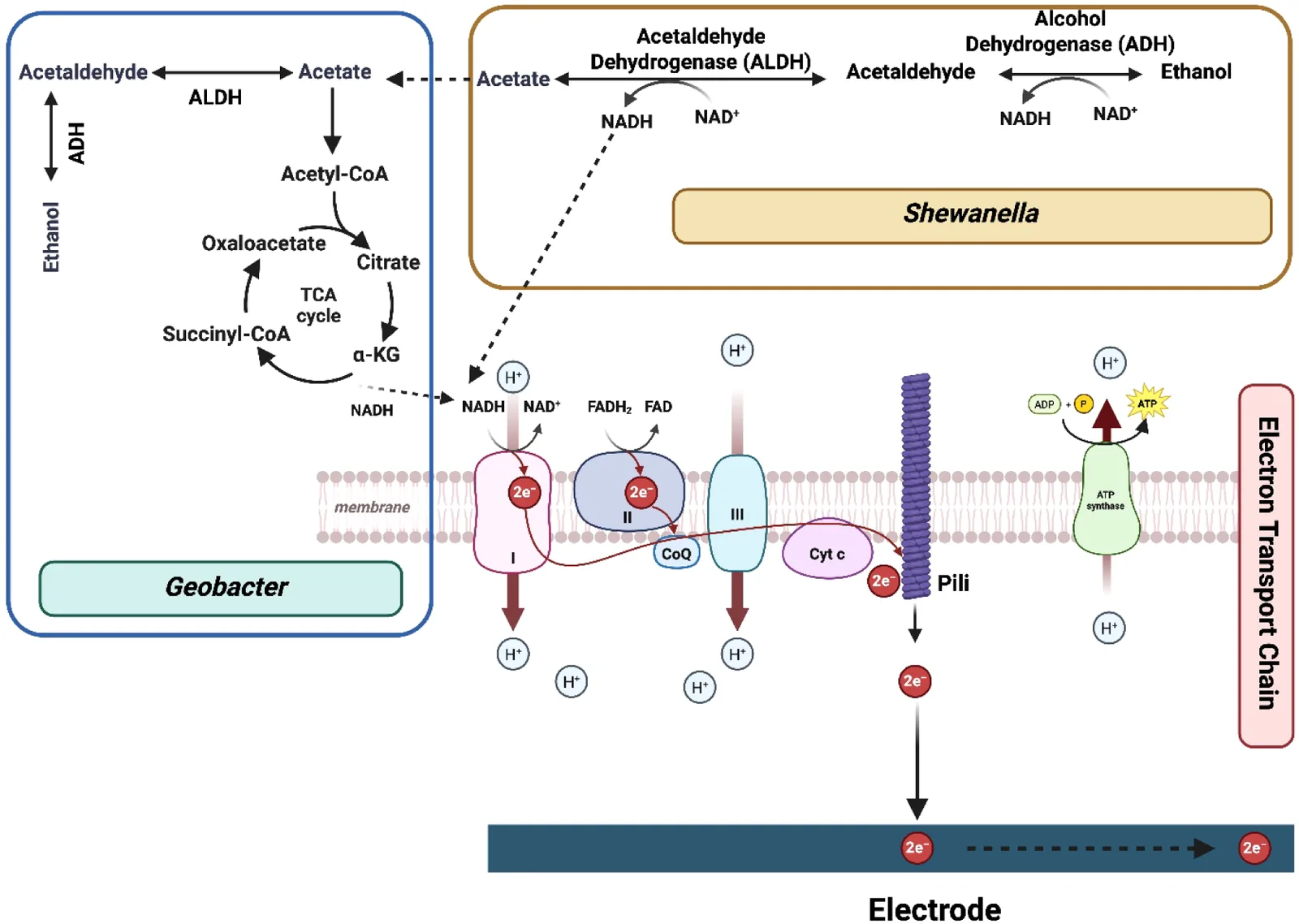
Proposed pathway of ethanol oxidation. Ethanol consumption (GC) and acetate formation (HPLC, ^1^H NMR) were measured.

Taken together, the electrochemical, chromatographic, spectroscopic, and sequencing data support the following working hypothesis for the enhanced performance of GM+SO bioanodes. Ethanol would first be oxidized to acetaldehyde by alcohol dehydrogenase (EC 1.1.1.1), which was predicted at similar abundance in SO and GM+SO (∼99,000–101,000 units). Acetaldehyde is oxidized further to acetyl-CoA via acetaldehyde dehydrogenase (acetylating; EC 1.2.1.10) and to acetate via the combined action of acetate kinase (EC 2.7.2.1) and phosphate acetyltransferase (EC 2.3.1.8). Aldehyde ferredoxin oxidoreductase (EC 1.2.7.5), prominently represented in GM and GM+SO (127,000 and 103,000 units, respectively), provides an alternative oxidation route that channels reducing equivalents directly to the ferredoxin pool, from which they are which could ultimately be passed to the outer-surface c-type cytochromes of *Geobacter* and to the anode.

## 4 Conclusion

This study showed that pairing *Geobacter metallireducens* with *Shewanella oneidensis* MR-1 on a single anode produced a bioanode that outperformed either organism alone across both acetate and a graded ethanol series. The hierarchy GM+SO > GM > SO held at every substrate condition over 82 days, and the coculture reached a peak current density of 1.66 ± 0.27 A m^–2^ at 5 mM ethanol while shifting its catalytic onset roughly 100 mV more negative than GM, which lowered the voltage needed to drive the same current. The clearest functional difference appeared at 20 mM ethanol, where ethanol removal after 96 h fell to 50% in GM but remained at 75% in GM+SO. Because the ethanol concentrations were applied sequentially, this decline may reflect substrate inhibition, biofilm aging, or both. HPLC, GC, and ^1^H NMR identified acetate as an intermediate of ethanol oxidation in all three configurations. CV and LSV showed that the coculture formed a bioanode with a larger catalytic response than either monoculture. At the end of operation, *Geobacter* and *Shewanella* accounted for 45% and 20% of 16S rRNA reads in the coculture biofilm.

These results support a working hypothesis in which both partners oxidize ethanol to acetate and *Geobacter* is mainly responsible for acetate oxidation and electron delivery to the anode. PICRUSt2 predictions of alcohol and aldehyde dehydrogenase and c-type cytochrome-related functions are compatible with this picture, but they describe genetic potential rather than activity. Flavin-mediated electron transfer is another possible contributor, but extracellular flavins were not quantified and their contribution cannot be evaluated from the present data. Medium-replacement voltammetry, extracellular flavin quantification, spatially separated cocultures, and transcriptomic or isotope-tracing studies would be needed to distinguish them.

Two practical lessons follow from this work. First, the advantage of coinoculation depended on substrate and loading and was clearest under ethanol at high loadings, so evaluating bioanodes on acetate alone may miss it. Second, the *S. oneidensis* bioanode removed ethanol rapidly but produced the least current, showing that substrate removal alone is a poor criterion for choosing partners in electroactive consortia. These findings are relevant to bioanode design for ethanol-rich effluents from distilleries, fermentation facilities, and biorefineries, although performance with real wastewater, hydrogen production, and energy efficiency remain to be evaluated.

## Supplementary Material



## Notes

Additional experimental information, including the composition of the modified Wolin’s mineral solution and Wolin’s vitamin solution used in DSMZ medium 579 (Table S1); ^1^H NMR spectra of reactor liquid from GM, SO, and GM+SO MECs (Figure S1); acetate formation in GM, SO, and GM+SO MECs over 96 h at 5, 10, 15, and 20 mM EtOH (Figure S2) (PDF)
