# Supplementary material for: Enhanced Ethanol Degradation Using Geobacter metallireducens – Shewanella oneidensis Coculture Bioanodes in Single-Chamber Microbial Electrolysis Cells
Source: ACS Omega. 2026 Oct 28;11(44):69348–61. doi: 10.1021/acsomega.6c10207 (PMC13756743; doi:10.1021/acsomega.6c10207)
Supplement: Supplementary file 1 [file ao6c10207_si_001.pdf]

Supplementary Information

Enhanced Ethanol Degradation Using *Geobacter metallireducens-Shewanella oneidensis* Co-Culture Bioanodes in Single-Chamber Microbial Electrolysis Cells

Avinash Jukanti[1], Vamshi Krishna Bommagani[1], Abhishiktha Chiliveru[1], Alex Schechter[2,3], Rivka Cahan[1,*]

[1] Department of Chemical Engineering, Ariel University, Ariel 40700, Israel;

[2] Department of Chemical Sciences, Ariel University, Ariel 40700, Israel;

[3] Research and Development Centre for Renewable Energy, New Technologies, Research Centre (NTC), University of West Bohemia, 30100 Pilsen, Czech Republic

* Correspondence: rivkac@ariel.ac.il (RC)

Additional experimental information, including the composition of the modified Wolin's mineral solution and Wolin's vitamin solution used in DSMZ medium 579 (Table S1); ^{1}H NMR spectra of reactor liquid from GM, SO, and GM+SO MECs (Figure S1); acetate formation in GM, SO, and GM+SO MECs over 96 h at 5, 10, 15, and 20 mM EtOH (Figure S2).

Table S1: Modified Wolin's mineral and vitamin solutions (DSMZ medium 579)

**Mineral solution**	**per L**	**Vitamin solution**	**per L**
Nitrilotriacetic acid ($C_6H_9NO_6$)	1.50 g	Biotin	20 mg
$MgSO_4 \cdot 7H_2O$	3.00 g	Folic acid	20 mg
$MnSO_4 \cdot H_2O$	0.50 g	Pyridoxine hydrochloric acid	100 mg
NaCl	1.00 g	Thiamine hydrochloric acid	50 mg
$FeSO_4 \cdot 7H_2O$	0.10 g	Riboflavin	50 mg
$CoSO_4 \cdot 7H_2O$	0.18 g	Nicotinic acid	50 mg
$CaCl_2 \cdot 2H_2O$	0.10 g	Calcium D-(+)- pantothenate	50 mg
$ZnSO_4 \cdot 7H_2O$	0.18 g	Vitamin B_{12}	1 mg
$CuSO_4 \cdot 5H_2O$	0.01 g	p-Aminobenzoic acid	50 mg
$AlK(SO_4)_2 \cdot 12H_2O$	0.02 g	(DL)-α-Lipoic acid	50 mg
H_3BO_3	0.01 g		
$Na_2MoO_4 \cdot 2H_2O$	0.01 g		
$NiCl_2 \cdot 6H_2O$	0.03 g		
$Na_2SeO_3 \cdot 5H_2O$	0.30 mg		
$Na_2WO_4 \cdot 2H_2O$	0.40 mg		

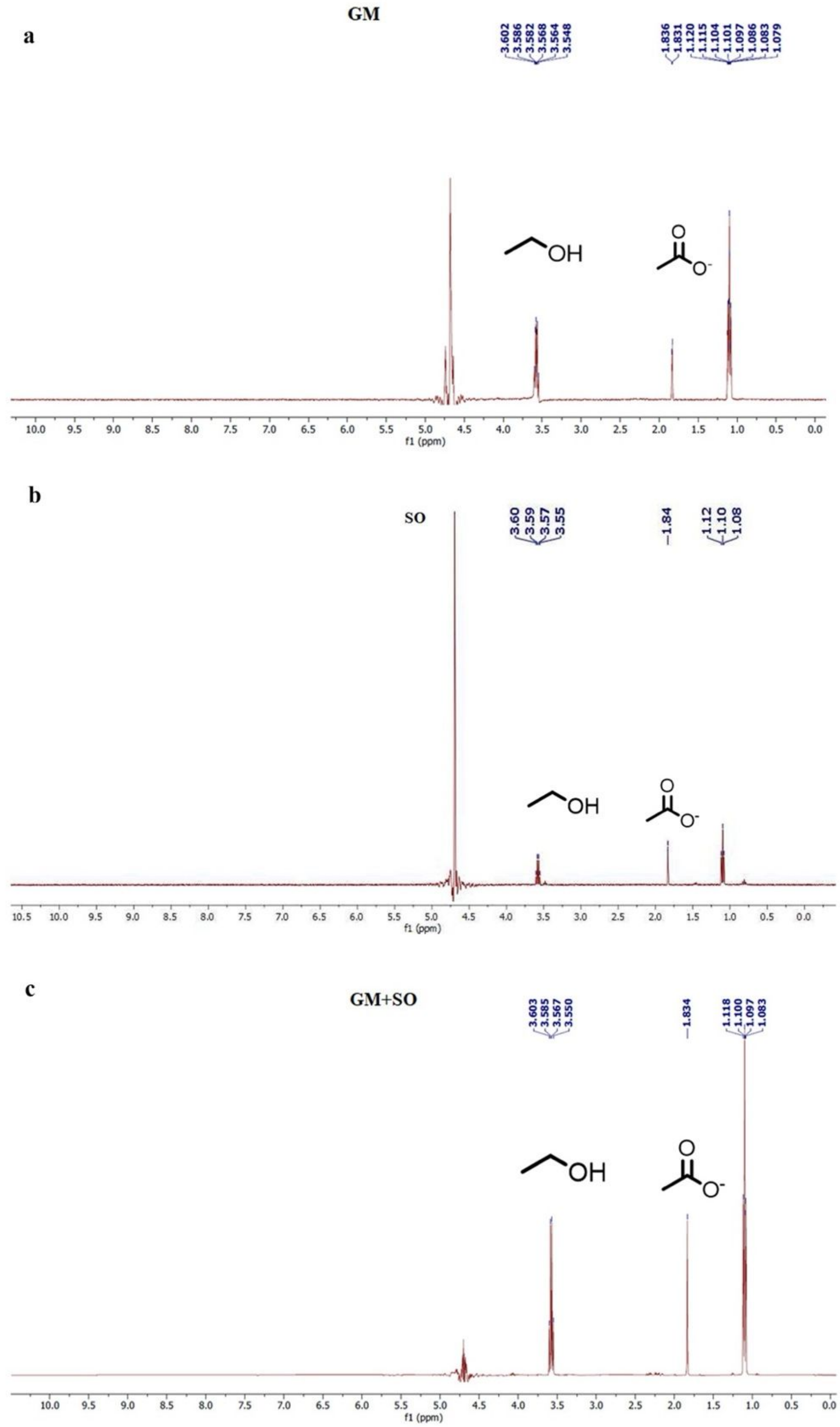

Figure S1. ^{1}H NMR spectra of (a) GM, (b) SO, and (c) GM+SO MECs after ethanol converted into acetate.

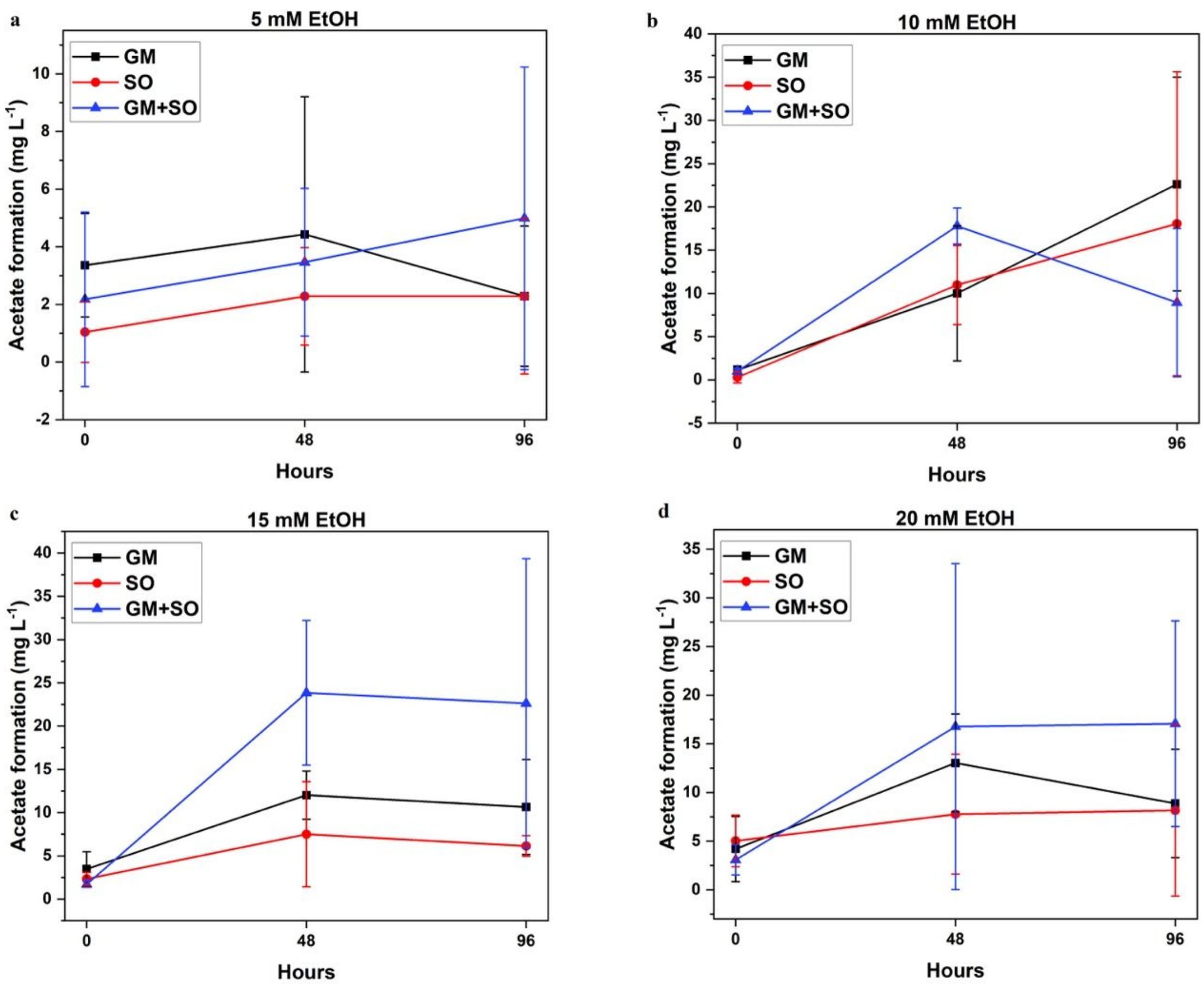

Figure S2. Acetate formation (mg L^{-1}) over 96 hours under increasing ethanol concentrations: (a) 5 mM, (b) 10 mM, (c) 15 mM, and (d) 20 mM EtOH.
